# Outcome of Pulmonary Large Cell Neuroendocrine Carcinoma After Definitive Treatment: A Single-Center Retrospective Review

**DOI:** 10.7759/cureus.86698

**Published:** 2025-06-24

**Authors:** Leone Sutanto, Ka Man Cheung, Chung Hang James Chow, Ho Yin Harry Yiu

**Affiliations:** 1 Department of Clinical Oncology, Queen Elizabeth Hospital, Kowloon, HKG

**Keywords:** cancer prognosis, chemotherapy, non-small cell lung cancer, pulmonary neuroendocrine carcinoma, targeted therapeutics

## Abstract

Introduction

Pulmonary large cell neuroendocrine carcinoma (LCNEC) is a rare and aggressive form of lung cancer. Given its poor prognosis and limited representation in clinical trials, the optimal treatment strategy remains undefined. This study aims to evaluate treatment modalities and clinical outcomes among LCNEC patients who received definitive treatment.

Methods

A retrospective review was conducted for patients diagnosed with LCNEC from 2000 to 2022 in a tertiary center. Clinical characteristics, staging, treatment modalities, molecular testing, and survival outcomes were collected. Progression-free survival (PFS) and overall survival (OS) were analyzed using Kaplan-Meier estimates.

Results

Twenty-seven patients were included. The median age was 69.6 years; most were male (92.6%) and smokers (92.6%). Distribution by stage was as follows: I (29.6%), II (22.2%), and III (48.1%). Surgery was performed in 77.8%, and 33.3% received chemoradiotherapy. Adjuvant platinum-etoposide chemotherapy was administered in 42.8% of surgical cases.

With a median follow-up of 75.9 months, the median PFS and OS were 9.6 and 20.8 months, respectively. The median OS by stage was as follows: I (30.2 months), II (23.5 months), and III (14.9 months). The recurrence rate was high (85.2%), with distant relapse being the most common (63%). Locoregional control was achieved in 40.7% of patients. Actionable mutations were detected in one patient (3.7%), and comprehensive molecular testing was underutilized.

Conclusion

LCNEC remains a clinically aggressive tumor with poor outcomes despite definitive treatment. The high recurrence rate, particularly in advanced stages, highlights the need for improved systemic strategies. Broader adoption of molecular profiling may uncover therapeutic targets and support the integration of emerging treatments, including immunotherapy.

## Introduction

Pulmonary large cell neuroendocrine carcinoma (LCNEC) represents a rare and highly aggressive subtype of pulmonary neoplasm, accounting for approximately 3% of all primary lung neoplasms diagnosed [1]. Compared to other forms of non-small cell lung carcinoma (NSCLC), LCNEC generally carries a worse prognosis and is highly fatal, regardless of the stage at diagnosis. Owing to its rarity, there remains a dearth of clinical data on prognostic factors and management strategies across early, locally advanced, and metastatic settings. Much of the current clinical practice is based on expert opinion and extrapolation from other types of pulmonary neoplasms.

In the past, LCNEC and small cell lung cancer (SCLC) were considered closely related, given that both represent high-grade forms of pulmonary neuroendocrine neoplasms, sharing common clinicopathologic characteristics such as aggressive behavior, a strong association with smoking history, male predominance, high proliferation rate, and generally poor prognosis [2]. In fact, a meta-analysis examining treatment regimens in both adjuvant and palliative settings suggested that LCNEC may derive greater survival benefit from SCLC regimens (e.g., etoposide with cisplatin) compared to NSCLC-based protocols.

In more recent years, however, it has been recognized that LCNEC is a distinct entity from SCLC, differing not only morphologically but also at the genomic and transcriptomic levels. Other major differences include a predilection for peripheral presentation in LCNEC (versus central in SCLC) and a higher proportion of early-stage diagnoses (25% vs. <5% in SCLC) [3].

Two major molecular subtypes of LCNEC have been described based on next-generation sequencing [4]. Type I (NSCLC-like) LCNECs are characterized by a lack of RB1 and TP53 co-alteration and frequent presence of NSCLC-type mutations (STK11, KRAS, KEAP1, NFE2L2). In contrast, type II (SCLC-like) LCNECs typically exhibit RB1 and TP53 co-alterations, absence of STK11 and KRAS mutations, and enrichment in alterations such as MYCL and SOX2 amplification and PTEN mutation or loss. While these genomic distinctions have been observed, whether they translate into meaningful clinical differences remains controversial due to conflicting data.

Given the rarity of LCNEC and the limited clinical evidence guiding its management, our study aims to fill this knowledge gap by conducting a retrospective cohort analysis focusing on outcomes in patients who received definitive treatment.

## Materials and methods

All consecutive cases of pathologically confirmed LCNEC from January 2000 to December 2022 were retrospectively identified from a single institutional lung cancer database. This single-center study was conducted at Queen Elizabeth Hospital, Kowloon, Hong Kong, in accordance with the principles of the Declaration of Helsinki. Institutional review board (IRB) approval was obtained from the Kowloon Central Cluster (KCC) Research Ethics Committee (approval number: KC/KE-23-0206/ER-1), and the requirement for informed consent was waived due to the retrospective nature of the study.

Inclusion criteria were age 18 years or above, pathological diagnosis LCNEC (either by biopsy specimen or from surgical specimen), and with an initial reception of definitive-intent treatment. Patients with incomplete medical records were excluded.

A total of 61 cases of pathologically confirmed LCNEC were initially identified. Three patients were excluded due to mixed histology with adenocarcinoma, five due to incomplete records, and the remainder as treatment was palliative-intent, yielding a final cohort of 27 patients treated with definitive intent.

In all eligible cases, clinical or pathological restaging was performed according to the International Union Against Cancer (UICC)/American Joint Committee on Cancer (AJCC) 8th Edition. The pathologic diagnosis of LCNEC was based on the microscopic appearance of the tumor cells, the mitotic rate, the proportion of intra-tumoral necrosis, and the presence of positive neuroendocrine markers identified by immunohistochemistry (IHC). The results of molecular testing for actionable mutations were reported if available.

Molecular testing was performed on available tumor tissue specimens to assess the presence of actionable oncogenic drivers. Epidermal growth factor receptor (EGFR) mutations were analyzed using polymerase chain reaction (PCR)-based assays targeting common sensitizing mutations in exons 18-21. IHC was used to evaluate anaplastic lymphoma kinase (ALK) and ROS1 protein expression. Positive IHC findings for ALK or ROS1 were defined based on strong granular cytoplasmic staining in tumor cells, as per institutional and guideline-based interpretation protocols. Confirmatory fluorescence in situ hybridization (FISH) was not routinely performed unless IHC results were equivocal. If available, next-generation sequencing (NGS) was conducted using targeted gene panels to detect a broader range of somatic mutations and gene fusions. PD-L1 expression was evaluated by IHC using the 22C3 pharmDx assay (Dako) [5], with tumor proportion score (TPS) reported as a percentage of tumor cells showing membranous staining.

Patient characteristics, including age, sex, and smoking status, were evaluated. Treatment modalities, including surgery, radiation details, and chemotherapy schemes, were explored. For surgically treated patients, adjuvant chemotherapy was defined as systemic therapy administered postoperatively, either as a standalone treatment or in combination with postoperative radiotherapy.

Statistical analyses were performed using IBM SPSS Statistics for Windows, Version 30.0 (Released 2019; IBM Corp., Armonk, New York, United States). Patient demographics and treatment details were summarized descriptively. The median follow-up was estimated using the reverse Kaplan-Meier method. Overall survival (OS) was measured from the date of diagnosis to death. Progression-free survival (PFS) was calculated from the start of treatment to disease progression or death. The first radiologic response was defined as the first post-treatment imaging assessment, typically performed after 2-3 cycles of systemic therapy or following the completion of radiotherapy. PFS and OS were estimated by the Kaplan-Meier method, censoring at the follow-up date if there was no radiological/clinical progression or death. Survival outcomes between subgroups (e.g., stage, treatment modality) were compared using log-rank tests. Prognostic factors were explored by univariate Kaplan-Meier and log-rank analysis. Multivariate analysis was not performed due to the limited cohort size.

## Results

A total of 27 eligible LCNEC patients were identified within the study period. Patient's demographics and treatments are summarized and shown in Table 1. The median age at diagnosis was 69.6 years (range: 52.6-81). The male-to-female ratio was 25:2. Most patients were smokers (92.6%). All patients were of Asian ethnicity.

**Table 1:** Patient demographics and treatment details ^a^: T-stage and N-stage were assigned according to the American Joint Committee on Cancer (AJCC) 8th Edition staging

Characteristics	n	(%)
Age, median (IQR)	69.6 (52.6-81)	
Sex		
Male	25	92.6
Female	2	7.4
Smoking status		
Never smoker	1	3.7
Smoker	25	92.6
Unknown	1	3.7
T-stage^a^		
T1	9	33.3
T2	10	37
T3	7	25.9
T4	1	3.7
N-stage^a^		
N0	12	44.4
N1	2	7.4
N2	10	37
N3	3	11.1
Actionable mutations		
Yes	1	3.7
No	10	37
Not tested	16	59.3
Primary treatment		
Surgery	21	77.8
Chemo-irradiation	9	33.3
Both	4	14.8
Adjuvant chemotherapy (post-surgery)		
Yes	9	42.8
No	12	57.1

In terms of cancer stage stratification at presentation, the numbers with stages I, II, and III (AJCC 8th Edition) were 8 (29.6%), 6 (22.2%), and 13 (48.1%), respectively. Treatment summaries of each patient are shown in Table 2.

**Table 2:** Individual patient treatment summary CRT: chemoradiotherapy; FU: follow-up; N/A: not applicable; RT: radiotherapy; LR: locoregional; SRT: stereotactic radiotherapy; IV: intravenous; NSCLC: non-small cell lung carcinoma; LCNEC: large cell neuroendocrine carcinoma; CR: complete response; PD: progressive disease †: The patient developed a second primary malignancy; no relapse of LCNEC was observed Chemotherapy regimens: atezolizumab: 1200 mg every 21 days; EP: etoposide 100 mg/m^2^ IV days 1-3 and cisplatin 75 mg/m^2^ IV day 1; EJ: etoposide 100 mg/m^2^ IV days 1-3 and carboplatin AUC 5/6 IV day 1; VJ: vinorelbine 30 mg/m^2^ IV days 1 and 8 and carboplatin AUC 5/6 IV day 1 Radiotherapy was generally delivered using conventionally fractionated NSCLC-type protocols; full-dose and fractionation details were not consistently available across the study period.

No.	Stage	Primary treatment	First response	Relapse pattern	Salvage	First treatment on relapse	Last seen	Survival to FU (months)
1	IA	Surgery	CR	LR		Symptomatic care	Dead	50.9
2	IA	Surgery	CR	Local	√	Radiotherapy (39 Gy/10 Fr)	Dead	28.7
3	IA	Surgery	CR	LR	√	Radiotherapy (40 Gy/10 Fr)	Dead	32.2
4	IB	Surgery	CR	N/A		Symptomatic care†	Dead	78.8
5	IB	Surgery	CR	Distant		Symptomatic care	Dead	11.5
6	IB	Surgery	CR	Distant	√	Lobectomy	Alive	101.8
7	IB	CRT	CR	Distant		Symptomatic care	Dead	6.5
8	IB	Surgery	CR	N/A		Not applicable	Alive	63.8
9	IIB	Surgery	PD	Distant		Atezolizumab+EJ	Alive	19.9
10	IIB	Surgery	PD	Distant		Palliative RT to spine, no chemo	Dead	8.3
11	IIB	Surgery	CR	Distant		Symptomatic care	Dead	23.5
12	IIB	Surgery	CR	Distant		EJ	Dead	25.7
13	IIB	Surgery	CR	Distant		Palliative RT	Dead	35.6
14	IIB	Surgery	PD	Distant		EJ	Dead	7.5
15	IIIA	Surgery and CRT	SD	Regional		On monitoring	Alive	16.6
16	IIIA	CRT	PD	Distant		Symptomatic care	Dead	14.8
17	IIIA	Surgery	PD	Distant		Whole brain RT	Dead	14.9
18	IIIA	Surgery	CR	Distant		Symptomatic care	Dead	14.1
19	IIIA	Surgery	PD	Distant		Symptomatic care	Dead	11.5
20	IIIA	Surgery and CRT	CR	Distant		EJ	Dead	16.5
21	IIIA	Surgery and CRT	PD	Distant		Erlotinib	Dead	23.9
22	IIIB	Surgery	CR	N/A		Not applicable	Alive	11.4
23	IIIB	Surgery and CRT	PD	Local		Symptomatic care	Dead	8.7
24	IIIB	CRT	CR	Distant	√	Craniotomy and SRT	Dead	46.8
25	IIIB	CRT	PR	Distant		Symptomatic care	Dead	10.5
26	IIIB	CRT	PR	Regional		EJ	Dead	20.8
27	IIIC	CRT	CR	N/A		Not applicable	Dead	12.3

The distribution of treatment modalities by stage is summarized in Table 3. Surgery alone was most frequently used in stage I and II disease (75% and 100%, respectively), whereas chemoradiotherapy (CRT) and combined-modality treatment were more common in stage III. However, the association between disease stage and treatment modality was not statistically significant (Fisher's exact test; p=0.125). Among patients treated with combined surgery and CRT (n=4), treatment sequencing was heterogeneous: two patients received postoperative CRT, one received neoadjuvant CRT followed by surgery, and one received salvage CRT after limited resection for diagnostic purposes.

**Table 3:** Distribution of treatment modality by disease stage

Stage	Surgery only	CRT only	Surgery+CRT	Total (n)
I	6 (75%)	1 (12.5%)	1 (12.5%)	8
II	6 (100%)	0 (0%)	0 (0%)	6
III	5 (38.5%)	5 (38.5%)	3 (23.1%)	13
Total	17 (63%)	6 (22.2%)	4 (14.8%)	27

**Figure 1:**
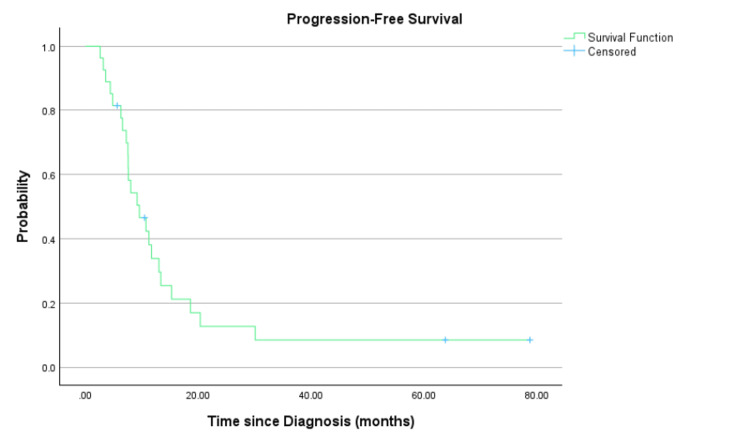
Progression-free survival of the overall population

**Figure 2:**
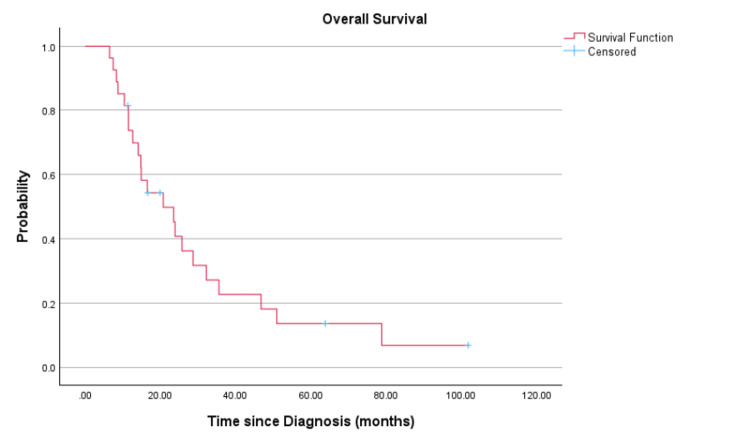
Overall survival of the overall population

At a median follow-up duration of 75.9 months (range: 6.5-101.7 months), the median PFS (Figure 1) and OS (Figure 2) of the whole cohort were 9.6 months (95% CI: 5.9-13.4 months) and 20.8 months (95% CI: 10.8-30.8 months), respectively.

**Figure 3:**
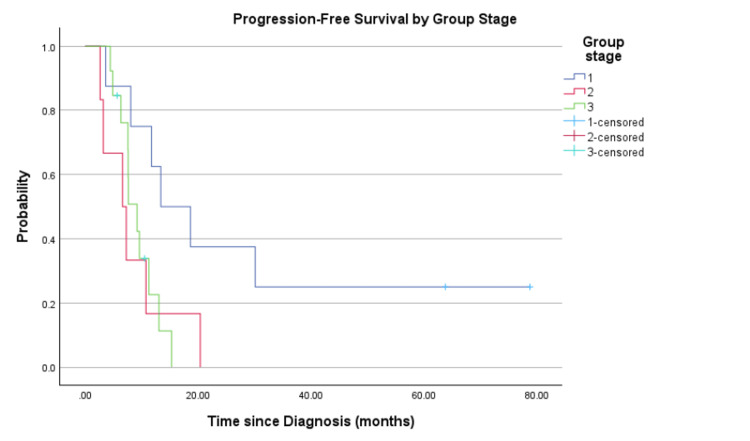
Progression-free survival by group stage

**Figure 4:**
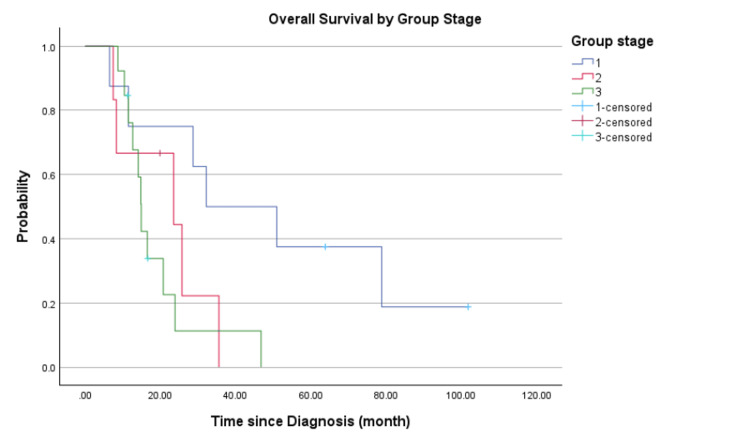
Overall survival by group stage

In the subgroup analysis, for stages I, II, and III, the PFS are 13.4, 6.6, and 9.2 months, respectively (Figure 3). The OS for these subgroups is 30.2, 23.5, and 14.9 months, respectively (Figure 4). Both PFS and OS differed significantly by disease stage, with lower-stage patients demonstrating improved outcomes (log-rank p=0.042 and p=0.040, respectively).

**Figure 5:**
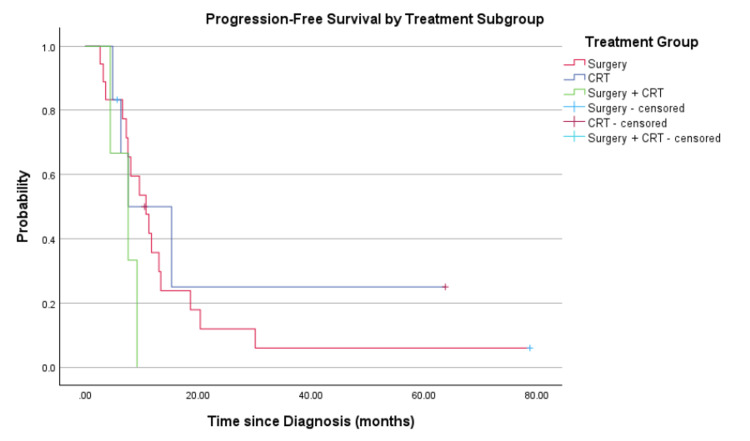
Progression-free survival by treatment subgroup CRT: chemoradiotherapy

**Figure 6:**
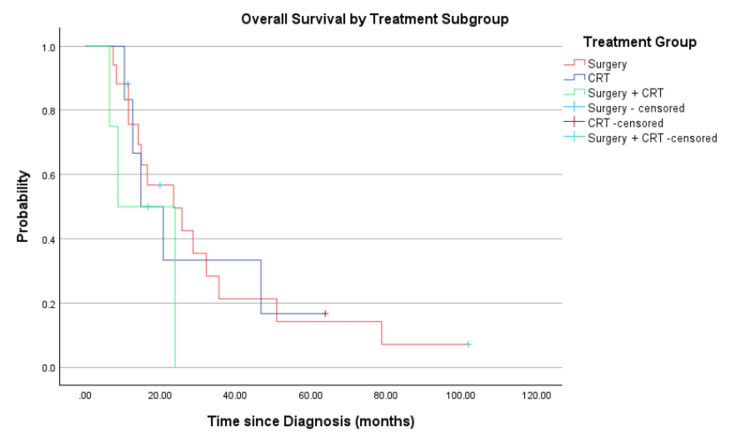
Overall survival by treatment subgroup CRT: chemoradiotherapy

A Kaplan-Meier analysis was performed to compare PFS and OS between treatment subgroups: surgery alone, CRT alone, and combined surgery with CRT (Figures 5-6). The median PFS was 10.6 months (surgery alone), 6.6 months (CRT alone), and 5.9 months (surgery+CRT). The corresponding median OS was 25.7 months (surgery alone), 14.1 months (CRT alone), and 16.5 months (surgery+CRT). While the difference in PFS approached statistical significance (log-rank p=0.061), no significant difference in OS was observed between the groups (log-rank p=0.534). Nonetheless, patients treated with surgery alone demonstrated numerically superior survival outcomes across both endpoints.

Among surgically treated patients (n=21), nine patients (42.8%) received postoperative chemotherapy. This included six patients who received adjuvant chemotherapy alone and three patients who received combined postoperative chemoradiation (chemotherapy with postoperative radiotherapy). Among patients treated with surgery alone (n=17), six (35.3%) received adjuvant chemotherapy, while 11 (64.7%) did not. The Kaplan-Meier analysis did not demonstrate a statistically significant difference in OS between patients who received adjuvant chemotherapy and those who did not (log-rank p=0.791). The median OS was 28.7 months (95% CI: 6.6-50.8) in the non-chemotherapy group, compared to 23.5 months (95% CI: 8.9-30.1) in the adjuvant chemotherapy group. These findings suggest limited observed benefit from adjuvant chemotherapy in this cohort, although interpretation is constrained by small sample size and limited statistical power.

For the majority of patients, disease progression or relapse is a common occurrence (85.2%). The overall locoregional control rate from definitive treatment was 40.7% (11/27). For patients with locoregional recurrence, four patients (25%) received salvage treatments, with only one patient (6.25%) remaining in remission after salvage treatment on long-term follow-up.

The predominant pattern of relapse was distant metastasis (63%), followed by regional (37%) and local recurrence (33%). A Cox regression analysis did not identify a statistically significant association between site of metastasis and OS. Brain (p=0.470), pleural (p=0.391), and adrenal (p=0.571) metastases were not independently associated with poorer survival outcomes. Bone and liver metastases were excluded due to collinearity or lack of variability in the dataset.

Due to the low uptake of immunotherapy in this cohort, meaningful statistical comparisons could not be performed.

Of note, less than half of the patients had actionable mutation testing for their pathological specimens. Of those who were evaluated, one patient tested positive for a druggable mutation and subsequently went on to receive EGFR kinase inhibitors upon progression with erlotinib.

## Discussion

Non-metastatic LCNEC accounts for less than half of all initial presentations [6]. Despite the use of definitive treatment, outcomes remain poor, with median survival ranging from eight to 12 months [2], though more favorable outcomes have been reported in some surgical series, with median survival reaching up to 36 months [7]. In comparison, outcomes for stage‑matched NSCLC are considerably more favorable: five‑year OS rates are around 47% for stage I, ~30% for stage II, and approximately 10% for stage III disease [8]. LCNEC clearly demonstrates more aggressive biology and poorer survival, even when similar treatment strategies are applied.

Treatment strategies in our cohort aligned with those described in prior studies. Most existing literature has focused on surgical outcomes, with limited data on non-surgical approaches [7,9]. In the absence of prospective trials, LCNEC management is typically extrapolated from NSCLC and SCLC protocols [2]. Recent reviews have emphasized the biologic heterogeneity of LCNEC and proposed molecular classification into SCLC-like and NSCLC-like subtypes, with implications for treatment selection [2,10].

Adjuvant chemotherapy was underutilized in our surgical cohort, consistent with patterns observed in other real-world studies [11]. This also reflects the evolving recognition of the importance of adjuvant chemotherapy compared to historical practice. Although no statistically significant survival benefit was observed in our cohort, this may be attributable to the limited sample size. Retrospective studies have reported improved survival with platinum-based adjuvant chemotherapy, particularly in node-positive or higher-stage LCNEC [12,13]. Given the tumor's aggressive nature, adjuvant therapy should remain a key consideration in suitable surgical candidates, especially in cases where the primary tumor is ≥3 cm [14].

In our series, patients with more advanced disease were more likely to receive definitive chemoradiation. No patients received maintenance immunotherapy. While prospective data in LCNEC are lacking, post-definitive immunotherapy is now supported in related histologies, including PACIFIC (NSCLC) [15] and ADRIATIC (SCLC) [16]. For resected NSCLC, adjuvant immunotherapy is standard for selected high-risk patients based on IMpower010 [17] and PEARLS/KEYNOTE-091 [18].

The optimal treatment strategy for resectable stage III LCNEC remains undefined. The ESPATUE trial [19], although not specific to LCNEC, included some patients with LCNEC in its study population and demonstrated comparable outcomes between surgery and chemoradiation in resectable stage III NSCLC, suggesting both may be reasonable options in selected cases.

Immunotherapy has also shown promise in the palliative setting, with reported improvement in median OS from 9 to 26.4 months in LCNEC [20]. More recently, a real-world retrospective series suggested that immunotherapy used as consolidation therapy in stage III LCNEC may confer a survival benefit [21]. Whether this benefit truly extends to post-definitive settings remains unknown, but extrapolation may be appropriate in selected cases, particularly those with NSCLC-like molecular features.

Previous studies have reported that actionable mutations, such as EGFR mutations or ALK/ROS1 fusions, are present in approximately 5-20% of LCNEC cases, though prevalence varies depending on cohort composition and molecular testing methods [22]. In our cohort, one patient was identified with an EGFR mutation and received erlotinib with a durable response. Molecular profiling was not routinely performed in earlier cases, as molecular testing was not standard clinical practice at the time. Furthermore, testing on archival blocks older than 20 years is subject to limitations related to tissue preservation and nucleic acid degradation, which may affect result reliability.

Our study's small sample size reflects the rarity of LCNEC and the inherent challenges in conducting large-scale trials; as such, comparisons of PFS and OS between treatment groups are exploratory in nature, as formal adjustment for patient baseline characteristics was not feasible. The evolving treatment landscape, including advances in systemic therapy, radiotherapy, and staging, further complicates long-term interpretation. Nonetheless, the extended follow-up and detailed patient-level data in our cohort offer meaningful insights into real-world outcomes for this uncommon disease.

## Conclusions

LCNEC remains a rare yet clinically aggressive malignancy with consistently poor outcomes, even after definitive treatment. Its high recurrence rate, particularly in advanced stages, as suggested by prior studies, reinforces the need for more effective systemic strategies. Our findings contribute to the growing body of real-world evidence highlighting the underutilization of adjuvant therapy and the emerging potential of immunotherapy in selected patients. Moving forward, the routine incorporation of molecular profiling, as increasingly recommended in current practice, may help clarify biologic subtypes and better inform treatment selection, including the rational use of targeted agents and immunotherapy. Given the absence of prospective trial data, collaborative multicenter registries and harmonized clinical protocols will be essential to accelerate progress and refine management of this understudied and lethal entity.
